# Hypoxia, ROS, and HIF Signaling in I/R Injury: Implications and Future Prospects

**DOI:** 10.3390/antiox15020153

**Published:** 2026-01-23

**Authors:** Manish Kumar Singh, Hyeong Rok Yun, Jyotsna S. Ranbhise, Sunhee Han, Sung Soo Kim, Insug Kang

**Affiliations:** 1 Department of Biochemistry and Molecular Biology, School of Medicine, Kyung Hee University, Seoul 02447, Republic of Korea; manishbiochem@gmail.com (M.K.S.);; 2 Biomedical Science Institute, Kyung Hee University, Seoul 02447, Republic of Korea; 3 Department of Biomedical Science, Graduate School, Kyung Hee University, Seoul 02447, Republic of Korea

**Keywords:** cardiomyocytes, ischemia, hypoxia, mitochondria, oxidative stress

## Abstract

Ischemic heart disease (IHD) remains a leading cause of morbidity and mortality worldwide. Myocardial ischemia–reperfusion injury (MIRI) is a significant contributor to cardiac tissue damage, resulting from an abrupt reduction in blood flow that leads to a reduction in the supply of oxygen and nutrients. The resulting hypoxia triggers severe cellular injury and impairs organ function. Hypoxia-inducible factors (HIFs) play a central role in maintaining oxygen homeostasis in mammalian tissues. As primary oxygen sensors, HIFs trigger the transcriptional activation of a wide range of genes that facilitate cellular adaptation to reduced oxygen availability and assist in minimizing ischemic damage. Mitochondria are particularly vulnerable to hypoxic stress and are a major source of reactive oxygen species (ROS) during I/R injury. Stabilization of HIFs has been shown to reduce loss of cardiomyocytes under these conditions, highlighting the importance of HIF-dependent pathways in preserving mitochondrial integrity and promoting cell survival. Collectively, these observations suggest that hypoxia, HIF signaling, and mitochondrial dysfunction are tightly interconnected processes in the pathogenesis of IHD. This review, therefore, focuses on the interaction between hypoxia-driven HIF responses and mitochondrial regulation, emphasizing their implications for therapeutic strategies in managing IHD.

## 1. Introduction

**Figure 1:**
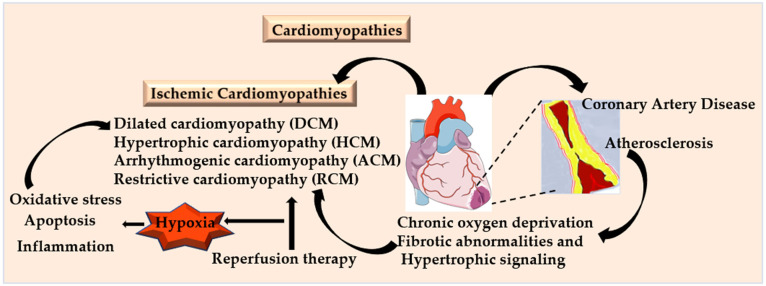
Schematic illustration of the pathogenic link between coronary artery disease, hypoxia, and cardiomyopathies. Atherosclerosis-induced coronary artery disease leads to chronic oxygen deprivation (hypoxia), triggering oxidative stress, apoptosis, and inflammation. These molecular events promote maladaptive cardiac remodeling, including fibrotic abnormalities and hypertrophic signaling, ultimately contributing to the development of ischemic cardiomyopathies such as dilated (DCM), hypertrophic (HCM), arrhythmogenic (ACM), and restrictive cardiomyopathy (RCM). Reperfusion therapy partially restores oxygen supply but may also exacerbate oxidative injury and hypoxia-associated damage.

Ischemic heart disease (IHD), also known as coronary artery disease (CAD) or coronary heart disease (CHD), comprises a heterogeneous group of pathological conditions characterized by an imbalance between myocardial oxygen supply and nutrient availability. This leads to insufficient blood flow and oxygen delivery to cardiac tissue and peripheral organs. Cardiomyopathies are generally classified into several types: ischemic cardiomyopathy, dilated cardiomyopathy (DCM), hypertrophic cardiomyopathy (HCM), arrhythmogenic cardiomyopathy (ACM), and restrictive cardiomyopathy (RCM). Ischemic cardiomyopathy (IC) usually develops as a result of chronic myocardial ischemia caused by CAD and atherosclerosis (Figure 1) [1]. In contrast, non-ischemic forms such as DCM, HCM, ACM, and RCM are frequently associated with genetic factors and can also be influenced by comorbidities such as hypertension [2]. The 2023 American guidelines have revised the binary classification of myocardial ischemic syndrome (MIS), which encompasses both obstructive and non-obstructive CAD. It is further subdivided into acute myocardial ischemic syndromes (AMIS) and non-acute myocardial ischemic syndrome (NAMIS), reflecting the various clinical presentations of acute and chronic angina, as well as myocardial ischemia [3]. Myocardial ischemia reperfusion injury (MIRI) refers to the paradoxical damage that can occur when blood flow is restored after a period of ischemia. Although reperfusion is widely used as a treatment for IHD, it may worsen myocardial injury due to oxidative stress, inflammation, and disruption of cell survival pathways. The aberrant activation of pathways involved in fibrosis and hypertrophy further contributes to the progression of disease [4]. Ultimately, cardiomyopathy from any cause can lead to congestive heart failure (HF), which is a significant global health challenge and a leading cause of morbidity and mortality [5,6]. According to the World Health Organization (WHO), CHD accounted for approximately 7.25 million deaths, representing 12.8% of all deaths, in 2008 [7]. In the United States, the annual economic burden of HF is projected to approach nearly USD 70 billion by 2030 [8].

The increasing prevalence of non-communicable diseases is largely driven by population aging, urbanization, and significant lifestyle changes, especially in low- and middle-income countries undergoing rapid socioeconomic transition [9]. Key contributing factors include high-calorie diets, less physical activity, obesity, insulin resistance, and type 2 diabetes mellitus (T2DM) [7,10]. Notably, men in South Asia and the Middle East carry a disproportionately high burden of these chronic conditions [11]. This epidemiological shift marks a global transition from communicable to noncommunicable diseases as the primary health threats. Atherosclerotic narrowing of the epicardial coronary arteries continues to be the main cause of myocardial ischemia [12]. Reduced coronary blood flow often results from intravascular thrombosis or progressive stenosis. A prolonged or complete interruption of coronary blood flow can lead to irreversible death of cardiomyocytes, a condition that is clinically referred to as myocardial infarction (MI). In some cases, reperfusion may worsen myocardial injury due to mechanisms associated with MIRI [12,13]. During ischemia, oxygen deprivation impairs the function of the endothelial barrier by inhibiting adenylate cyclase activity and reducing intracellular cAMP levels, which increases vascular permeability [14]. Both ischemia and reperfusion activate multiple cell death pathways, including apoptosis, autophagy, and necrosis.

Early signs of atherosclerosis often present as lesions that cause a reduction of less than 50% luminal diameter of coronary arteries, which significantly increases proximal resistance and decreases distal coronary perfusion pressure. While autoregulation can maintain basal coronary blood flow, the capacity for dilation is compromised. As a result, patients may remain asymptomatic at rest but experience inadequate blood flow during periods of high metabolic demands, such as physical exercise [15]. However, as stenosis progresses, it limits oxygen delivery when myocardial oxygen demand increases, leading to symptoms such as angina pectoris [16], HF, arrhythmias, thrombosis, embolism, dyspnea, and pulmonary edema [17,18]. Although angina is most commonly associated with CAD, it can also occur in other conditions such as valvular heart disease, HCM, or uncontrolled hypertension [16]. In rare cases, angina may persist even when coronary angiography shows normal results; this can be attributed to coronary vasospasm or microvascular dysfunction [19,20,21]. Coronary spasm in the presence of atherosclerotic lesions can induce MI, with the degree of response related to the plaque burden [22]. Additionally, patients experiencing focal coronary vasospasm may display non-significant atherosclerosis on angiography [23], indicating that vasospasm can develop in both the early and advanced stages of the disease. Reperfusion therapy is crucial in managing ischemic cardiomyopathy, but hypoxia and HIFs also exhibit significant protective roles in restoring blood flow and minimizing further damage from MI [24]. Consequently, signaling pathways associated with hypoxia, mitochondrial reactive oxygen species (mtROS), and HIFs have emerged as important therapeutic targets in IHD [25].

Furthermore, atherosclerosis involves dyslipidemia, inflammation, and oxidative stress as part of the immune response [26,27]. Studies have reported various circulating biomarkers for oxidative stress, including malondialdehyde (MDA), oxidized LDL (ox LDL), and 8-isoprostanes (F_2_-IsoPs) directly derived from lipid oxidation, while ferric-reducing antioxidant power (FRAP), superoxide dismutase (SOD), and glutathione (GSH) are indirectly associated with lipid oxidation [28]. Among these, MDA is the most evaluated lipid biomarker and is identified as an independent predictor of CVD risk factor in patients’ biological samples, including plasma and urine [29,30,31,32]. This review aims to provide a comprehensive and up-to-date synthesis of the roles of hypoxia, mitochondrial ROS (mtROS), and HIF signaling in myocardial ischemia and reperfusion (I/R) injury.

## 2. Hypoxia and Regulation of HIF Activity

**Figure 2:**
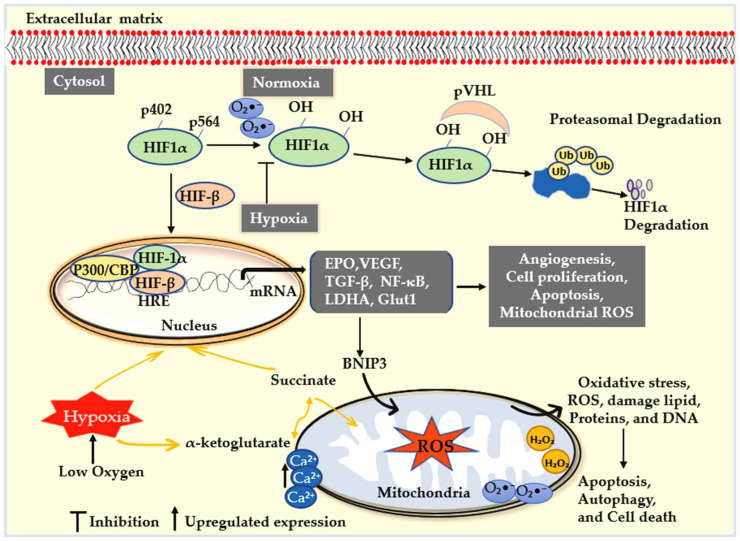
Schematic illustration of hypoxia-inducible factor-1α (HIF-1α) regulation and downstream signaling under normoxic and hypoxic conditions. Under normoxia, HIF-1α undergoes hydroxylation and proteasomal degradation via the pVHL pathway, whereas hypoxia stabilizes HIF-1α, enabling its nuclear translocation and transcriptional activation of target genes involved in angiogenesis, metabolism, cell survival, and mtROS production. Calcium overload and BNIP3 induce mitochondrial dysfunction, leading to apoptosis and cell death.

Hypoxia is defined as a lack of sufficient oxygen to sustain normal cellular activities. This condition disrupts energy homeostasis and initiates a cascade of molecular events that can impair cardiac function. HIFs are evolutionarily conserved transcription factors that play an essential role in assisting metazoans adapt to an environment with reduced oxygen availability [33]. Although Porifera (sponges) and Ctenophora (comb jellies) can tolerate low or even anoxic (no oxygen) conditions without relying on the HIF pathway, these exceptions illustrate that certain species possess HIF-independent mechanisms [34]. In mammalian systems, however, HIF activity is tightly regulated by oxygen levels. Under normoxic conditions, HIFs are quickly degraded through the proteasomal pathway. In contrast, during hypoxic conditions, HIF protein becomes stabilized and activates the transcription of a wide range of target genes, including erythropoietin (EPO), glucose transporter 1 (GLUT1), vascular endothelial growth factor-A (VEGF-A) [35], multiple glycolytic enzymes [36], and enzymes involved in generating extracellular adenosine (Figure 2) [37].

HIFs are heterodimeric transcription factors made up of an oxygen-regulated α-subunit (HIF1α) and a constitutively expressed β-subunit (HIF1β) [38], also known as the aryl hydrocarbon receptor nuclear translocator (ARNT) [39]. The α-subunit comprises three main isoforms: HIF-1α, HIF-2α, and HIF-3α [40]. Among these, HIF-1α, identified by Semenza and colleagues in 1991, is the most extensively studied and plays a central role in coordinating the cellular response to hypoxia [41]. HIF-1α contains two transactivation domains: N-terminal domain (TAD-N; amino acids 531–575) and a C-terminal domain (TAD-C; amino acids 786–826). Both domains are responsible for recruiting transcriptional coactivators such as CBP, p300, SRC-1, and TIF-2 [42]. The intervening region (amino acids 576–785) serves as an inhibitory domain, which is modulated by oxygen levels, independent of the stability of HIF-1α protein [42]. Factor-inhibiting HIF-1 (FIH-1) interacts with the C-terminal region of HIF-1α (amino acids 757–826), which includes the inhibitory domain and TAD-C. This interaction fine-tunes the transcriptional activity of HIF-1α in response to varying oxygen levels [43]. Interestingly, the three HIFα isoforms have distinct functional roles that depend on tissue and cell specificity. Despite some structural similarities, HIF isoforms exhibit different or even opposing activities [44]. For instance, HIF-1α selectively regulates the inducible nitric oxide synthase (iNOS) gene (NOS2), while HIF-2α regulates the expression of arginase 1, resulting in divergent effects on macrophage polarization and cancer metastasis [45,46]. Familial cases in humans have shown that erythrocytosis is associated with a gain-of-function mutation in the HIF-2α gene at Gly537 [47], or with an inactivating mutation in the PHD2 gene [48]. In contrast, HIF-3α does not have a functional transactivation domain and is generally considered an inhibitory element, thus also known as inhibitory PAS protein (IPAS) [49]. Compared to HIF-1α and HIF-2α, the role of HIF-3α, along with its various isoforms and splice variants, is less clearly defined.

Hypoxia is crucial for I/R injury, which leads to a limited supply of metabolites and oxygen, triggering coronary artery occlusion, and, if prolonged, causes myocardial cell death and subsequent heart failure [12,50,51]. The reduced availability of oxygen prevents mitochondrial oxidative phosphorylation, resulting in reduced ATP production and impaired contractility of the heart muscle cells. During myocardial ischemia and reperfusion (I/R), HIF-1α provides cardioprotective effects, in part, by activating the purinergic signaling pathway that enhances myocardial resistance to ischemic stress. These pathways primarily involve A2A and A2B adenosine receptors [52]. In the cardiovascular system, extracellular purinergic signaling is regulated by ectonucleoside triphosphate diphosphohydrolase-1 (NTPDase1/CD39), a key enzyme that hydrolyzes ATP to produce adenosine. While this pathway plays a vital role in maintaining vascular homeostasis, excessive accumulation of extracellular purine metabolites has been associated with various metabolic and cardiovascular diseases [53]. Additionally, hypoxia affects the activity of ion channels, causes calcium overload, and triggers maladaptive signaling pathways that can worsen myocardial injury. The most significant impact of the hypoxic response is the inhibition of oxygen-sensing enzymes, the prolyl hydroxylase domain (PHD), at specific residues (Pro 402 and Pro 564) within the O_2_-dependent degradation domain (ODDD) [54]. These results suggest that HIF-PHD(s) serve as critical sensors of oxygen levels, regulating the HIF transcriptional cascade [55].

The HIF signaling pathway is connected to the enzymatic activity of HIF-PHDs. PHDs are part of the Fe (II)- and 2-oxoglutarate-dependent oxygenase superfamily, which requires molecular oxygen for their enzymatic activity [55,56]. There are three isoforms of HIF-PHD: PHD1 (EGLN2), PHD2 (EGLN1), and PHD3 (EGLN3) [57]. Under normoxia, PHD1 and PHD2 promote the hydroxylation of HIF-α at specific proline residues: Pro564 (HIF-1α), Pro530 (HIF-2α), and Pro490 (HIF-3α). This hydroxylation process facilitates recognition by the E3 ubiquitin ligase complex that includes the von Hippel–Lindau (pVHL) protein. This interaction leads to ubiquitination and rapid proteasomal degradation of HIF-1α [33,58] (Figure 2). Additionally, HIF-1α stability is also influenced by p53 protein, which recruits the E3 ligase MDM2, providing another layer of hypoxia-dependent regulation [59]. In contrast, hypoxic conditions facilitate the hydroxylation of proline residues, which stabilizes HIF-1α and promotes its translocation into the nucleus, dimerization with HIF-β, and transcriptional activation of genes involved in angiogenesis, cell survival, metabolic reprogramming, and vascular remodeling (Figure 2).

FIH1 is another Fe (II)- and 2-oxoglutarate-dependent dioxygenase that hydroxylates a conserved asparagine residue within the C-terminal transactivation domain (CTAD) of HIF-α [43,60]. This modification prevents the recruitment of coactivators p300/CBP under normoxic conditions [61,62]. FIH1 remains active at lower oxygen levels than PHDs, thereby restraining HIF-α activity even under moderate hypoxia [63,64]. Notably, HIF-2α is more resistant to hydroxylation by FIH-1 than HIF-1α, suggesting that there are isoform-specific activation thresholds and differential utilization of the CTAD [65,66].

### 2.1. Hypoxia-Associated Signaling Pathways

**Figure 3:**
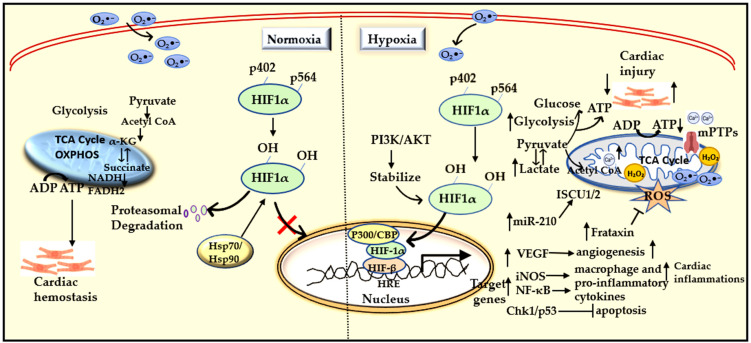
Schematic illustration of HIF-1α signaling and ROS upon low oxygen (hypoxia). The two panels represent the differential regulation of HIF-1α signaling and cellular metabolism under normoxic and hypoxic conditions in cardiomyocytes. Under normoxia (left panel), HIF-1α is hydroxylated at proline residues (P402 and P564) in the presence of oxygen, targeting it for proteasomal degradation via the pVHL pathway. Mitochondrial oxidative phosphorylation (OXPHOS) predominates, with pyruvate entering the tricarboxylic acid (TCA) cycle to generate ATP, thereby maintaining cardiac homeostasis. In contrast, under hypoxia (right panel), reduced oxygen availability inhibits HIF-1α hydroxylation, leading to its stabilization through PI3K/AKT signaling and chaperone-mediated activity. Stabilized HIF-1α translocates into the nucleus, dimerizes with HIF-1β, and recruits transcriptional coactivators (p300/CBP) to activate hypoxia response element (HRE)-containing target genes. This results in enhanced glycolysis, lactate production, angiogenesis (VEGF), inflammatory signaling (iNOS, NF-κB), and metabolic reprogramming via miR-210 and ISCU1/2. Hypoxia-induced mitochondrial dysfunction promotes reactive oxygen species (ROS) generation, mitochondrial permeability transition pore (mPTP) opening, calcium dysregulation, and ATP depletion, collectively contributing to cardiac injury, inflammation, apoptosis, and pathological remodeling. Red (X) indicates prevent HIF 1α translocation into the nucleus.

Hypoxia plays a significant role in various biological processes, including metabolic reprogramming, tumor progression, cardiomyopathy, arrhythmogenesis, angiogenesis, and neurodegenerative disorders. Through the modulation of gene expression, hypoxia impacts the clinical and pathological characteristics of numerous human diseases. Central to the cellular response to low oxygen levels is the activation of HIF transcription factors, which orchestrate metabolic adaptation, regulate cell proliferation, and modulate inflammatory responses, among other functions [67]. In MIRI, initial hypoxia promotes AKT phosphorylation and the subsequent activation of HIF-1α. This process promotes the induction of several cardioprotective genes (Figure 3) [68]. Hypoxia also intersects with multiple intracellular signaling pathways, including phosphoinositide 3-kinase (PI3K), mammalian target of rapamycin (mTOR), nuclear factor-κB (NF-κB), extracellular signal-regulated kinases (ERK), toll-like receptor (TLR) signaling, and endoplasmic reticulum (ER) stress pathways. PI3K/AKT and protein kinase A (PKA) signaling are particularly important for HIF stabilization and enhancing its transcriptional activity [24]. Pharmacological enhancement of AKT signaling increases VEGF expression, promoting angiogenesis and limiting tissue damage after reperfusion. Additionally, AKT activation protects cardiomyocytes by inhibiting apoptosis induced by hypoxia or oxidative stress via hydrogen peroxide (Figure 3). Interestingly, mechanical stimuli such as pressure overload and hypoxia can activate endogenous AKT, providing acute protection to cardiac cells [69]. This apparent paradox suggests that while acute AKT activation may offer protective benefits, chronic or sustained activation may fail to prevent progressive myocardial dysfunction and could even contribute to adverse cardiac remodeling [70]. HIF-α isoforms have different effects in inflammatory cells. In macrophages, HIF-1α and HIF-2α play antagonistic roles in regulating nitric oxide (NO) homeostasis. iNOS operates downstream of HIF-1α and has a cardioprotective effect during MIRI.

Other factors such as hemeoxygenase-1 (HO-1), adiponectin, insulin-like growth factor-2, and GLUT are involved in the protective effects of HIF-1α against MIRI [71]. In contrast, HIF-2α, which is enriched in anti-inflammatory M2 macrophages, suppresses NO synthesis via the arginase-1 pathway [72]. The switch between HIF-α isoforms modulates NO levels and influences vascular functions. Increased levels of HIF-1α are also linked with lower systemic blood pressure, whereas elevated HIF-2α is linked to hypertension [73]. Moreover, during oxidative stress, HIF-1α activates the Nrf2 antioxidant pathway, enhancing the expression of enzymes that clear cellular ROS. However, hypoxia-induced inflammation is exacerbated through the activation of NF-κB signaling and characterized by the phosphorylation and degradation of IκBα [74,75]. HIF-1α can also inhibit NF-κB signaling and induce HO-1, which reduces the production of pro-inflammatory cytokines, lessens tissue inflammation, and ultimately mitigates the severity of MIRI [68]. Inhibition of PHDs during hypoxia triggers post-translational signaling cascades that stabilize both HIF and the inflammatory transcription factor NF-κB. This interplay between hypoxic and inflammatory pathways is significant in cardiovascular disease (Figure 3) [50].

Previous studies using experimental models to manipulate PHD protein expression have demonstrated the central role of HIF signaling in ischemia and reperfusion. In vivo studies on PHD3 knockout mice show that the loss of this protein exacerbates HIF1-α stabilization while reducing the activation of DNA damage response pathways mediated by ATR, Chk1, and p53 [76]. Collectively, these changes mitigate myocardial I/R injury by reducing myocyte apoptosis [77]. Thus, reducing PHD3 expression enhances cardiac ischemic tolerance [78]. Conversely, overexpression of PHD3 in hypoxic tissues impairs HIF-α accumulation and blunts the adaptive response to ischemia, even though it does not alter baseline cardiac function in resting animals [79]. Similarly, the loss of PHD2 enhances cardiac function and reduces infarct size, further highlighting the protective role of HIF stabilization in acute myocardial infarction [80].

### 2.2. Hypoxia Associated Post-Translational and Epigenetic Modulations

HIF-α stability is further modulated by various post-translational pathways. Inhibition of heat shock protein 90 (HSP90) and the use of histone acetylase inhibitors can promote HIF-α degradation independently of pVHL. Additionally, SUMOylation has effects on HIF-α stability that depend on the specific context [39,81]. In vivo studies showed that SUMOylation generally destabilizes HIF-α; for instance, the deletion of the deSUMOylating enzyme SENP1 results in impaired HIF activation and anemia [82]. Interestingly, SUMO1 is induced by hypoxia, suggesting a feedback mechanism where SUMOylation dynamically adjusts the hypoxic response [83,84]. Several histone deacetylases (HDACs) have been reported to enhance HIF-α stability and accumulation by regulating the interaction between HIF-α and PHD2. Notably, HDAC4, HDAC5, and HDAC6 improve HIF-1α stability and transcriptional activity by modulating the acetylation status and function of crucial HIF-1α cofactors, such as HSP90 and p300 [85,86,87]. Inhibition of either HDAC4 or HDAC5 reduces HIF-1α protein levels and suppresses its activity, indicating that these HDACs play a role in the post-translational regulation of HIF-1α through modulation of the HSP70/HSP90 chaperone machinery (Figure 3) [85].

Moreover, hypoxia drives epigenetic remodeling beyond acetylation. In human cardiac fibroblasts, prolonged exposure to hypoxia induces a pro-fibrotic phenotype characterized by global DNA hypermethylation and increased expression of DNMT1 and DNMT3B [88]. Additionally, hypoxia-regulated microRNAs have been linked to clinicopathological features and clinical outcomes across multiple cancer types. For instance, hypoxia activates AKT signaling, leading to increased expression of miR-21 in an NF-κB and CREB-dependent manner [89]. During hypoxia, HIF-1α induces miR-210, which targets mitochondrial genes iron-sulfur cluster enzyme 1/2 (ISCU1/2) (Figure 3) [90], thereby reducing the activity of iron-sulfur-dependent enzymes, such as aconitase and mitochondrial complex 1 [91]. Additionally, miR-199a acts as a negative regulator of HIF-1, linked with SNP rs2057482, which correlates with a reduced premature CVD risk but increased cancer susceptibility [92,93,94]. Despite these insights, the epigenetic and post-transcriptional landscapes of hypoxia in MIRI remain largely unexplored and require further investigations.

## 3. Mitochondrial Oxidative Stress and Regulation of Hypoxia

Studies have demonstrated that mitochondria play a crucial role in coordinating cellular responses to hypoxia. During hypoxia, mitochondria adjust metabolic pathways to maintain energy production, but simultaneously produce ROS [89]. Excessive generation of mtROS triggers oxidative stress, which can damage lipids, proteins, and mtDNA. These stress activates redox-sensitive transcription factors such as HIF-1α, NF-κB, and p53 [95,96]. This imbalance between ROS production and antioxidant defense compromises mitochondrial integrity, promotes apoptosis, and contributes to pathological cardiac remodeling [97]. During ischemia, reduced oxygen availability impairs mitochondrial oxidative phosphorylation, resulting in decreased ATP production and increased intracellular Ca^2+^ accumulation [98]. This calcium overload, combined with oxidative stress, can lead to mitochondrial dysfunction and cause cardiomyocyte apoptosis or necrosis during MIRI. Elevated levels of ROS and Ca^2+^ can induce the opening of mitochondrial permeability transition pore (mPTPs), which are highly conductive and nonselective. When activated, mPTPs cause oxidative modifications to mitochondrial proteins and lipids, ultimately triggering cardiomyocyte death [99].

Under hypoxic conditions, oxygen-dependent inhibition of PHD enzymes stabilizes HIF-1α. HIF stabilization regulates mitochondria-specific genes that enhance mitochondrial function and promote cell survival [100]. For instance, HIF-1α upregulates frataxin, a mitochondrial protein that alleviates iron overload and ROS production, thereby preserving mitochondrial membrane integrity and cardiomyocyte viability (Figure 3) [101]. HIF-1α also enhances mitochondrial respiration through cardioprotective pathways, including PI3K/AKT and JAK2/STAT3 signaling, which provide myocardial protection during I/R injury [102]. Conversely, mitochondria also regulate the stability of HIF-1α: ROS-derived electron transport chain can promote HIF-1α accumulation in hypoxic conditions, supporting mitochondrial function and membrane integrity while enhancing cardiomyocyte survival during I/R injury. This bidirectional regulation highlights the complex interplay between mtROS production, oxygen availability, and HIF-1α signaling [103].

### 3.1. Mitochondrial Oxidative Stress and HIF Regulation

**Figure 4:**
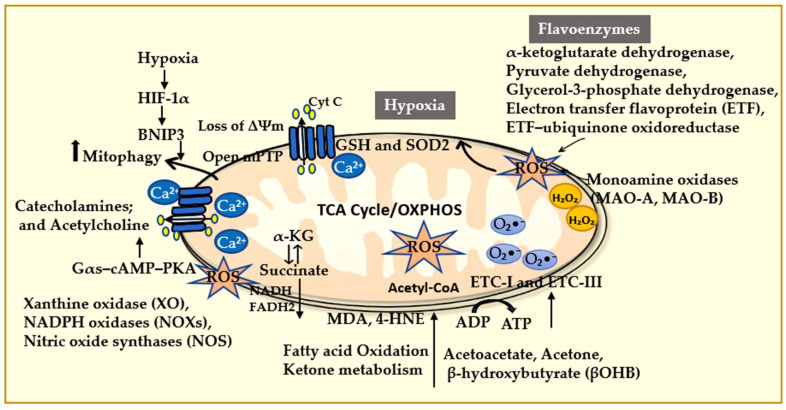
Schematic illustration of the interplay between hypoxia, HIFs, and mtROS. Under hypoxic conditions, stabilization of HIFs alters mitochondrial metabolism and enhances oxidative stress from multiple sources. In addition to electron transport chain (ETC) complexes I and III, mitochondrial stress is generated during fatty acid oxidation, lipids, and ketone body metabolism, and through non-ETC mitochondrial enzymes, including α-ketoglutarate dehydrogenase, pyruvate dehydrogenase, glycerol-3-phosphate dehydrogenase, electron transfer flavoprotein (ETF), and ETF–ubiquinone oxidoreductase. Other cellular oxidant systems, such as xanthine oxidase (XO), NADPH oxidases (NOXs), uncoupled nitric oxide synthase (NOS), and monoamine oxidases (MAO-A/B), further amplify mtROS production, particularly at ETC complexes I and III. Elevated oxidative stress exerts detrimental effects, including oxidative damage to lipids, proteins, and DNA. Antioxidant defense mechanisms counterbalance oxidative stress through upregulation of cytoprotective molecules such as glutathione (GSH) and superoxide dismutase 2 (SOD2); genetic impairment of these systems exacerbates oxidative injury in vivo. Succinate signaling modulates catecholamine and acetylcholine pathways via Gαs–cAMP–PKA signaling, leading to Ca^2+^ release and enhanced mitochondrial activity. HIF-1α-mediated induction of BNIP3 promotes mitophagy as an adaptive response to hypoxia.

Early studies in *S. cerevisiae* demonstrated that the induction of hypoxic response genes and the suppression of aerobic genes depend on an intact mitochondrial respiratory chain, which highlights the mitochondria as central regulators of oxygen-sensitive transcriptional programs [104]. The expression of these gene networks is highly sensitive to precise oxygen concentrations, underscoring the critical role of mitochondrial oxygen sensing in cellular adaptation to hypoxia. In mammalian myocardium, mitochondria supply the majority of cellular energy, generating approximately 6 kg of ATP per day in humans through OXPHOS [105,106]. Under low oxygen levels, the AMP/ATP ratio becomes the primary regulator of oxidative phosphorylation, activating AMP. This activation quickly adjusts cardiac energy metabolism, enhancing glucose uptake and fatty acid oxidation to maintain ATP production by phosphorylating several key metabolic enzymes, without significant changes in thermodynamic driving forces [107,108]. Persistent oxidative stress leads to excessive accumulation of mtROS, which triggers the opening of the mPTP [109]. mPTP opening destabilizes the mitochondrial membrane potential and initiates cascades of cardiomyocyte injury and apoptosis (Figure 4) [110,111]. These pathological processes mainly arise from oxidative damage to lipids, proteins, and nucleic acids. Under hypoxic conditions, increased ROS promotes lipid peroxidation of polyunsaturated fatty acids, resulting in the formation of lipid peroxides and their reactive aldehyde derivatives, including MDA and 4-hydroxy-2-nonenal (4-HNE). These toxic byproducts impair membrane integrity and fluidity, with the inner mitochondrial membrane being particularly susceptible to such damage due to the localized nature of the produced mtROS [95]. Cyclosporine A (CsA) is a drug widely used after organ transplants and has demonstrated a protective effect in animal models by inhibition of mPTP opening and subsequent cell death [112,113].

Studies have demonstrated that increased expression of HIF-1α activates its downstream effector, BNIP3, in response to I/R injury. The induction of BNIP3 promotes mitophagy, a mitochondria-dependent autophagy [114], which attenuates excess ROS accumulation and restores energetic homeostasis [115]. This selective removal of dysfunctional mitochondria is crucial for the survival of cardiomyocytes and serves as an important adaptive mechanism against hypoxic stress (Figure 4) [116]. Consequently, the HIF-1α/BNIP3 pathway represents a promising therapeutic target for treating hypoxia-induced myocardial injury. Supporting this concept, recent research has indicated that berberine (BBR) enhances mitophagy by activating the HIF-1α/BNIP3 pathway. This process facilitates the clearance of damaged mitochondria, reduces apoptosis, and promotes myocardial repair following I/R injury [117]. However, excessive mitochondrial dysfunction and elevated ROS levels can hinder autophagic progress by oxidatively modifying essential proteins involved in mitophagy [114]. Additionally, the activation of other stress responses, such as the unfolded protein response (UPR) and various forms of programmed cell death-like apoptosis, necroptosis, and pyroptosis, competes for limited cellular energy resources [118]. This competition for resources becomes particularly pronounced during severe hypoxia, ultimately leading to decreased efficacy of mitophagy and worsening cardiomyocyte dysfunction and cell death [97,119].

### 3.2. Oxidative Stress and Mitochondrial Dysfunction in IHD

Numerous studies have demonstrated that the development of ischemic cardiomyopathy (ICM) is closely linked to disruptions in mitochondrial quality control. In cardiac disorders, both mitochondrial and extramitochondrial sources have been implicated in cardiac disorders, including I/R, HF, and arrhythmias. Under normal physiological conditions, cardiac mitochondria generate approximately 95% of cellular ATP through oxidative phosphorylation (OXPHOS), utilizing a variety of substrates, including fatty acids, glucose, lactate, ketone bodies, pyruvate, and amino acids, which are metabolized through the tricarboxylic acid (TCA) cycle and the electron transport chain (ETC) complexes I–V. The remaining ~5% of ATP is produced via anaerobic glycolysis [120,121]. Notably, mtROS are recognized as a major source of intracellular ROS, particularly during aging or mitochondrial dysfunction. In MIRI, ROS production begins during ischemia and significantly increases upon reperfusion, constituting a central driver of tissue injury. Moreover, ROS are generated through substrate-specific metabolic pathways. For instance, fatty acid oxidation and ketone metabolism also contribute to ROS production. Ketone bodies, such as acetoacetate, acetone, and β-hydroxybutyrate (βOHB), are synthesized in the liver, with βOHB being the predominant circulating form and an alternative energy substrate when glucose and fatty acid availability are limited [122]. The oxidation of βOHB in the heart produces ROS as a byproduct primarily through ETC complexes I and III, where superoxide (O_2_^•−^) is generated from premature electron leakage to molecular oxygen [123].

Mitochondrial myocardial enzymes, including NDUFV2, SDHA, Cyt b, Cox2, ATP5A-MC I-V, serve as key indicators of myocardial energetic function. Among metabolic intermediates, the accumulation of succinate is recognized as an early hallmark of hypoxia [124,125]. Mitochondrial oxidative processes are tightly interconnected with adrenergic and cholinergic signaling pathways. Succinate dehydrogenase (SDH) and its substrate, succinate, regulate the synthesis and release of catecholamines such as epinephrine and norepinephrine, while α-ketoglutarate is similarly associated with acetylcholine metabolism [126]. Sympathetic stimulation enhances mitochondrial performance through the canonical Gαs–cAMP–PKA signaling pathway [127], promoting intracellular Ca^2+^ release and augmenting mitochondrial activity. Succinate itself also modulates mitochondrial Ca^2+^ handling, further linking metabolic flux to mitochondrial stability (Figure 4) [128]. Hypoxia disrupts intracellular Ca^2+^ homeostasis, resulting in cytosolic Ca^2+^ overload that aggravates mitochondrial injury and induces endoplasmic reticulum stress. This form of stress activates pro-apoptotic pathways involving CHOP and caspase-12, accelerating cardiomyocyte loss [96]. In vivo studies have shown that diltiazem, a calcium-channel antagonist, combined with SOD, exhibits cardioprotective effects against MIRI. These findings suggest that mitigating Ca^2+^ levels may reduce oxidative stress and inhibit apoptosis, minimize cardiac injury caused by MIRI, and delay the onset of HF [129].

### 3.3. Role of Mitochondrial-Targeted Antioxidants in Hypoxia Regulation

Mitochondrial-targeted antioxidants (MTA) such as MitoQ, elamipretide, SkQ1 [130], CoQ10 [131], and melatonin [132] play a crucial role in mitigating oxidative damage by stabilizing mitochondrial membrane potential and inhibiting the opening of mPTP. These reagents exhibit antioxidant properties by reducing lipid peroxidation, lowering calcium ion concentration [133], and preventing apoptosis, thereby preserving cardiac and renal functions [134,135]. Specifically, MitoQ restores Sirt-3 expression and mitochondrial homeostasis, which helps alleviate renal I/R injury both in vivo and in vitro, highlighting its critical role in oxidative stress-related diseases [136]. Elamipretide protects cardiolipins from ROS, maintains membrane integrity, reduces calcium ion entry, prevents apoptosis, and ATP depletion [137]. Similarly, SkQ1 neutralizes superoxide anions, stabilizing the inner membrane structure and maintaining the function of ETC [130]. CoQ10 acts as an antioxidant, neutralizes free radicals, and inhibits the excessive mPTP opening and inhibits apoptosis [138]. Furthermore, it exerts anti-inflammatory effects by reducing the release of inflammatory cytokines and mitigating cell damage caused by chronic inflammation [139]. Melatonin, an endogenous hormone secreted by the pineal gland, acts as a potent free radical scavenger, alleviating the effects of free radicals and reactive nitrogen species, thereby reducing oxidative stress-induced cellular damage [140]. It also has modulatory effects on blood vessels and the heart through its interaction with membrane receptors MT1, Mel1A, MTNR1A, MT2, Mel1B, MTNR1B, and the retinoid-related orphan nuclear receptors RZR and RORα [141]. Studies have demonstrated its protective role in reducing the risk of heart failure and post-infarction cardiomyopathy [140,141,142]. These findings suggest MTA holds significant potential for improving I/R injury.

The mitochondrial H_2_O_2_ scavenging, which regulates both matrix and cytoplasmic H_2_O_2_ levels, depends on NADPH and the recycling of thioredoxin (Trx) and GSH. Subsequently, two GSH molecules are oxidized by H_2_O_2_ through GPX, generating oxidized glutathione (GSSG) [143]. GSSG is subsequently reduced back to GSH by glutathione reductase (GSR1) using NADPH. Since mitochondria cannot synthesize GSH or export GSSG, this reduction is strictly limited by compartmentalized mitochondrial NADH/NADPH production. Consequently, a coordinated redox network between the cytoplasm and mitochondria is crucial for cardiac ROS scavenging [144]. Mitochondria can activate adaptive responses to hypoxia, but they are also vulnerable to oxygen deprivation. Hypoxia exacerbates oxidative stress and mitochondrial injury, disrupting metabolic homeostasis and worsening cellular dysfunction [145]. As an O_2-_senstive transcription factor, HIF-1α orchestrates key metabolic adaptations to hypoxia. However, prolonged or chronic hypoxia increases the risk of ischemic ventricular arrhythmias and promotes cardiomyocyte death [146]. Identifying optimal oxygen levels that preserve mitochondrial function while maintaining protective HIF-1α activity could unveil new therapeutic strategies for mitigating MIRI [24].

Several non-ETC enzymes significantly contribute to mtROS. Flavoenzymes, including α-ketoglutarate dehydrogenase, pyruvate dehydrogenase, glycerol-3-phosphate dehydrogenase, electron transfer flavoprotein (ETF), and ETF-ubiquinone oxidoreductase, serve as major sources of mtROS [147]. Under certain metabolic states, these enzymes can produce more superoxide than the ETC [148]. Additionally, outer mitochondrial membrane-associated monoamine oxidases (MAO-A and MAO-B) generate hydrogen peroxide (H_2_O_2_) as a byproduct of neurotransmitter deamination, such as serotonin, which further exacerbates oxidative stress (Figure 4) [149]. Specifically, mtROS activate the Nrf2 antioxidant pathway, which upregulates cytoprotective molecules such as GSH and mitochondrial superoxide dismutase 2 (SOD2), thereby enhancing the cellular antioxidant defense system [150]. In vivo studies indicate that impaired SOD2 activity leads to mitochondrial structural damage, reduces ejection fraction, and results in a dilated, dysfunctional left ventricle, a key feature of dilated cardiomyopathy (Figure 4) [151]. For instance, the single Ala16Val MnSOD and Pro198Leu GPx polymorphism are associated with CHD susceptibility and severity. Notably, it has been found that the 16Val variant significantly increases CHD risk in men and contributes to CVD severity in a Tunisian population, correlating with reduced SOD2 activity, increased mtROS production, and endothelial dysfunction [152,153]. The GPx (C>T, *rs1050450*) variant is associated with decreased enzyme activity in T-allele carriers, as demonstrated in Caucasian cohorts with diabetes [154]. Consistently, a recent meta-analysis confirmed an association between the GPx1 variant and CAD in individuals with T2DM from Chinese and Indian populations [155]. Furthermore, dysregulation of the GSH-PX and PRX/Trx antioxidant pathways exacerbates oxidative stress, contributing to left ventricular (LV) contractile dysfunction and adverse remodeling, especially with angiotensin II stimulation [156]. Following I/R injury, these genetic predispositions may enlarge infarct size, impair cardiac function, and promote cardiomyocyte death [157,158].

Beyond mtROS, an extra-mitochondrial source of ROS production is driven by enzymes such as xanthine oxidase (XO), NADPH oxidases (NOXs), and uncoupled nitric oxide synthases (NOS), which interact with mtROS to intensify cellular oxidative stress (Figure 4) [103,159]. Conversely, endothelial NOS (eNOS)-derived NO is highly protective against MIRI. The activation of the PI3K/AKT and JAK2/STAT3 pathways [160,161] enhances eNOS activity and elevates the NO bioavailability to mitigate MIRI [162]. The Kelch-like ECH-associated protein 1(Keap1)/nuclear factor erythroid 2-related factor 2 (NRF2) signaling axis serves as a master regulator of this antioxidant defense. Oxidative stress disrupts the Keap1-Cul3 ubiquitin ligase complex, allowing Nrf2 to accumulate and translocate to the nucleus, where it triggers the expression of essential detoxifying antioxidant enzymes, such as HO-1, NAD(p)H: quinone oxidoreductase 1 (NQO1), SOD, Gpx, ferretin, and Trx system [147,163,164].

## 4. Interaction Between Hypoxia, ROS, and HIFs in IHD

ROS play a central role in ischemic-reperfusion injury (IRI) and are a major focus of mechanistic and therapeutic research. IRI represents a paradox where the restoration of blood flow to ischemic myocardium exacerbates tissue damage due to excessive ROS generation and cardiomyocyte apoptosis. A key driver of this process is the accumulation of succinate during ischemia, which fuels reverse electron transport at mitochondrial complex I upon reperfusion, resulting in a surge of ROS production [125]. The subsequent increase in ROS, combined with Ca^2+^ influx induced by reperfusion, promotes the opening of the mPTP. This further impairs ETC function and amplifies oxidative stress. HIFs, particularly HIF-1α and HIF-2α, contribute to myocardial IRI by modulating cellular metabolism and limiting mtROS production and apoptosis [125,165]. HIF-1α enhances mitochondrial integrity and enhances antioxidant defenses by activating the Nrf2 pathway, which upregulates key antioxidant enzymes such as GSH and SOD2 [36,150]. Additionally, HIF-1α promotes a metabolic shift toward glycolysis, reducing ETC activity and decreasing mtROS generation, thereby protecting cells from oxidative injury [166]. HIF-1α also modulates ROS derived from both mitochondria and NOXs, contributing to redox homeostasis and attenuating the severity of myocardial IRI [167]. Excessive mitochondrial Ca^2+^ loading and ROS accumulation together trigger persistent mPTP opening, leading to bioenergetic collapse and irreversible cardiomyocyte necrosis and apoptosis [168].

Pharmacologic stabilization of HIF-1α during reperfusion has been shown to attenuate myocardial IRI, underscoring the therapeutic potential of targeting HIF-dependent pathways. Experimental data also demonstrate that HIF-1α is a key mediator of ischemic preconditioning, further supporting its promise as a target for reducing myocardial I/R injury. However, HIF signaling is marked by context dependency. HIF regulates a broad array of genes essential for the survival and function of both cardiomyocytes and non-parenchymal cardiac cell types. Evidence from transgenic mouse models suggests that prolonged HIF activation can lead to HF [169,170,171], raising concerns that long-term administration of HIF-prolyl hydroxylase inhibitors may precipitate or exacerbate cardiac dysfunction. For instance, persistent HIF-1α expression in PHD2 knockout mice is associated with aggravated dilated cardiomyopathy [172]. In contrast, HIF-1α-mediated mitophagy via the HIF-1α/BNIP3 pathway supports the survival of acute cardiomyocytes after ischemic injury [116]. Long-term stabilization of HIF-1α may impair angiogenesis by favoring the expression of genes that inhibit cell cycle progression or cellular differentiation over proliferation [173]. Furthermore, prolonged HIF-1α activity disrupts mitochondrial homeostasis and metabolic pathways associated with energy homeostasis [67]. Additionally, chronic HIF-1α stabilization induces AMPK signaling, reactive gliosis, impaired visual function, ultimately amplifying metabolic stress and neurodegeneration in the retina [174].

During hypoxic stress, HIF-1α regulates the expression of lactate dehydrogenase A (LDH-A) and phosphoglycerate kinase 1 (PGK1), which facilitate a shift from oxidative phosphorylation to glycolysis. By suppressing mitochondrial respiration and enhancing glycolytic pathways, HIF-1α provides cellular adaptation and cell survival [175]. Furthermore, HIF-1α activates cardioprotective signaling pathways, including PI3K/AKT and JAK2/STAT3, which improve mitochondrial respiratory function and mitigate ischemic injury in part by increasing frataxin expression and protecting against iron overload [101]. Collectively, these findings suggest that the stabilization of HIF-1α confers protection in IHD by promoting cellular and metabolic adaptation to hypoxia during I/R. It is essential to further explore the distinct and overlapping roles of HIF-1α and HIF-2α to develop safe and effective therapies against HIF proteins that aim to reduce IRI while minimizing long-term adverse effects on the cardiac cells.

Another key modulator, Sirtuins, plays a crucial role in macrophage polarization through metabolomic and inflammatory signaling pathways in I/R injury. For instance, Sirt1 protects cardiomyocytes from oxidative stress by deacetylating and activating PGC-1α and FOXO, thereby upregulating antioxidant enzymes catalase and MnSOD [176]. Although its expression decreases in the heart following I/R, conversely, its overexpression enhances functional recovery by increasing MnSOD and Trx1, and anti-apoptotic protein Bcl-xL, while suppressing pro-apoptotic protein Bax [177]. Pharmacological activators such as resveratrol and nicotinamide mononucleotide (NMN) attenuate myocardial injury via modulating ERK and p38/JNK signaling [178], and restoring autophagic flux, resembling ischemic preconditioning through the NAMPT-mediated pathway [179]. Sirt5 exerts bidirectional control over macrophage polarization by regulating glycolytic control. It promotes the desuccinylation of pyruvate kinase M2 (PKM2), stabilizing its enzymatically active tetrameric form, a critical rate-limiting step of glycolysis [180].

Additionally, Sirt6 serves as a metabolic checkpoint linking glucose metabolism to inflammation and angiogenesis. It inhibits intracellular accumulation of succinate and destabilizes HIF-1α, thereby suppressing the release of interleukin (IL)-1β [181], and facilitates macrophage transition toward an anti-inflammatory phenotype. Through its defatty-acylase activity, Sirt6 stimulates VEGF secretion and promotes endothelial cell migration, capillary formation, and sprouting angiogenesis. Conversely, its genetic ablation impairs angiogenesis, whereas its overexpression restores vascular integrity and preserves cardiac function [182]. During hypoxia, Sirt6 stabilizes HIF-1α, induces VEGF expression, enhances angiogenesis, and reduces hemorrhage, contributing to anti-atherosclerotic effects [183]. In I/R injury, Sirt6 further mitigates oxidative stress via AMPK-dependent upregulation of antioxidant defenses [184]. Furthermore, comprehensive genetic analysis in North Chinese cohorts identified Sirt3 SNP rs28365927 (G>A), and Sirt6 SNPs rs350846 (G>C), rs107251 (C>T), and rs350844 (G>A) as key modulators of coronary artery disease susceptibility [185]. Collectively, these findings underscore a key mechanistic role of Sirt1, Sirt5, and Sirt6 in myocardial inflammation, metabolism, and vascular repair, highlighting sirtuin-targeted therapies as promising strategies for heart failure and ischemic heart disease [186,187].

## 5. Pharmacological Therapy for Hypoxia-Related Ischemic Heart Disease

### 5.1. Pharmacological Drugs’ Effects on I/R

HIF activation supports various adaptive processes, which include stimulating erythropoiesis, optimizing cellular metabolism under hypoxic conditions, and enhancing tissue resilience during ischemia, inflammation, and cancer [50,188,189]. Conversely, HIF stabilization has been explored therapeutically for retinal neovascularization [190], renal anemia [191], pulmonary hypertension, acute kidney injury [192], liver [193,194], and certain cancers. Sodium–glucose cotransporter 2 (SGLT2) inhibitors represent a relatively new class of antihyperglycemic drugs that offer significant cardiovascular benefits [195,196]. SGLT2is exhibit both direct and indirect effects on the cardiovascular system, significantly reducing the risk of cardiovascular disease. Notably, SGLT2is such as dapagliflozin and canagliflozin show a reduction in cardiovascular-related deaths, regardless of the patient’s diabetes status [197,198]. The direct effects of SGLT2i include inhibiting myocardial Na+/H+ exchange, improving myocardial metabolism, alleviating cardiac preload and afterload, and lowering blood pressure by osmotic diuresis [199]. They also help reduce cardiomyocyte apoptosis and improve myocardial fibrosis [200,201], reduce the synthesis of adipokines and cytokines [202], lower precordial adipose tissues [203], and attenuate sympathetic nerve activity [204]. Indirect effects include improving blood glucose levels, reducing glycosylated hemoglobin, and reducing the risk of hypoglycemia [205], promoting weight loss, regulating blood pressure [206], improving renal function by suppressing proteinuria [207], and delaying the progressive damage of diabetic nephropathy [208].

Dapagliflozin (DAPA) has been associated with reduced cardiovascular mortality and fewer HF exacerbations in diverse patient populations, including diagnosed HF [209,210]. DAPA has been shown to suppress myocardial fibrosis both in vitro and in vivo [211]. DAPA attenuates post-MI cardiac dysfunction through coordinated suppression of inflammation, cardiomyocyte apoptosis, and oxidative stress [211,212]. However, the precise therapeutic effects of DAPA on cardiac function after MI and the mechanism underlying these effects have not been fully elucidated. Another drug, PT2977 (belzutifan), prevents the transcriptional activity of HIF2α and inhibits its binding to HIF1β [213]. It is currently undergoing phase III clinical trials for the treatment of renal cell carcinoma in patients with VHL disease (Table 1) [214]. 3′-Methoxypuerarin (3′-MOP), a structural derivative of puerarin, exhibits significant cardioprotective effects in vitro and in vivo, by modulating m6A methylation and inhibiting pyroptosis [215].

Dihydrotanshinone I (DT) and protocatechuic aldehyde (PCA) have shown cardioprotective potential through mechanisms such as preconditioning and antioxidant activity [150,216]. DT temporarily induces ROS by reversibly inhibiting mitochondrial complex I, which stabilizes HIF-1α. The stabilized HIF-1α subsequently increases the transcription of Nrf2, enhancing the cellular antioxidant defenses [150]. PCA raises the levels of reduced GSH and increases reducing equivalents, improving ROS scavenging. Notably, sequential administration of DT followed by PCA significantly enhanced antioxidant capacity and protected cardiomyocytes against I/R injury, ultimately reducing infarct size and improving cardiac function in vivo [150].

Metformin is a first-line treatment for T2DM and also has significant cardioprotective effects. In rat models of MIRI, metformin reduces infarct size and lowers plasma lactate dehydrogenase and creatine kinase-MB. These benefits are achieved through the activation of AMPK signaling pathways, which suppress the expression of NOX4 and attenuate oxidative stress and apoptosis [217]. Dexmedetomidine (DEX) protects the heart from myocardial I/R injury by activating signaling pathways involving Erk1/2, AKT, and eNOS, while inhibiting inflammatory pathways. This leads to a smaller infarct size and improved cardiac function in both in vivo and ex vivo models [218]. DEX additionally decreases the expression of HIF-1α at the post-translational level and inhibits the transcriptional activation of its downstream effector, BNIP3 (Table 1). Administering DEX post-treatment protects against cardiac I/R injury in vivo and hypoxia/reoxygenation (H/R) injury in vitro [219]. Sevoflurane (SEV), a commonly used anesthetic agent, is preferred for its rapid onset and recovery, minimal airway irritation, and ease of maintenance during surgery. These characteristics have made SEV a standard choice in cardiac surgery, where it has demonstrated cardioprotective properties [220].

Roxadustat, a first-in-class oral HIF-2α stabilizer, enhances the production of EPO in renal anemia patients with chronic kidney disease (CKD), regardless of their dialysis status [221]. Phase III clinical trials of roxadustat (FG-4592) have been conducted in China for treating anemia in patients with low-risk myelodysplastic syndrome (MDS) [222], and it is approved in Japan for treating CKD based on four Phase III clinical studies [223]. In oxygen-induced retinopathy (OIR), roxadustat provides dual protection in premature children either through direct retinal HIF stabilization via aerobic metabolic shift or through indirect hepatic HIF-1 stabilization by enhanced secretion of hepatokines. Conversely, dimethyloxalylglycine (DMOG) provides only remote hepatic protection, successfully reducing I/R infarct size through HO-1 and EPO induction [224]. It lacks direct retinal protection through HIF stabilization and relies entirely on the hepatic-mediated pathway. The systemic synergy between the liver and other visceral organs has also been evident in hyperoxia-induced lung injury, where HIF stabilization in both the liver and lung prevents alveolar destruction [225]. The efficacy of the HIF stabilizer depends heavily on the timing of administration. While short-term early in-hospital infusions may mitigate cardiorenal and organ damage, their efficacy remains unclear. Conversely, post-discharge interventions have successfully improved cardiac function and quality of life [226]. Given that HF patients face a 30% annual mortality and a readmission rate of 25% within 30 days [227]. Several long-term oral and subcutaneous therapies are currently in clinical Phase II trials to assess their clinical utility [228].

Additionally, daprodustat, vadadustat, molidustat, and enarodustat are now approved in Japan [229], the European Union, the US, and other regions [230,231]. A meta-analysis confirms that these agents effectively raise hemoglobin (Hb) levels, with cardiovascular safety (observed at the time to the first major adverse cardiovascular event, MACE), and improved quality of life in both non-dialysis-dependent and dialysis-dependent CKD patients [232]. Specifically, roxadustat and daprodustat demonstrated comparable efficacy and cardiovascular safety (Table 1). A meta-analysis of 25 randomized controlled trials (RCTs) including 17,204 participants confirmed that agents lower LDL cholesterol and total cholesterol levels; however, they have not been shown to reduce the incidence of MACEs [233]. Furthermore, the long-term use of HIF-PHI treatment may increase thrombotic risk compared to erythropoiesis-stimulating agents (ESAs) [234,235], necessitating regular monitoring before and during the treatment [236].

Natural compounds have also been shown to modulate HIF-1α activity, thereby reducing MIRI. For instance, saponins from *Panax notoginseng* protect the HIF-1α/Bcl-2/BNIP3 axis, promoting mitochondrial autophagy [237]. β-Sitosterol has demonstrated lowering the blood cholesterol levels and alleviates MIRI induced cardiac dysfunctions through regulation of pyroptosis in rat [238]. Asiatic acid reduces ROS accumulation, enhances mitochondrial membrane potential, and decreases intracellular Ca^2+^ concentration, collectively improving mitochondrial function and decreasing myocardial damage [239]. However, a detailed mechanism behind these effects remains to be explored.

### 5.2. HIF-Targeted Gene and Cell-Based Therapy in MIRI

HIF overexpression via viral vectors offers a localized alternative to systemic stabilization, significantly mitigating off-target effects in cardiac and tumor models [240]. In critical limb ischemia (CLI), intramuscular injection of adenoviral expressing VEGF or HIF-1α (AdCA5) has been shown to reduce edema, improve perfusion, and improve quality of life (QOL) [241,242]. Similarly, HIF-1α overexpression in diabetic mice normalizes VEGF levels, improving glucose metabolism and angiogenesis [243]. However, the efficacy of gene therapy varies by condition; for instance, Ad2/HIF-1α/VP16 failed to significantly improve cardiac function in patients with peripheral artery disease (PAD) and intermittent claudication, highlighting the need for disease-specific optimization.

Stem cell-based therapies, including MSCs (mesenchymal stem cells), ADSCs (adipose-derived stem cells), EPCs (endothelial progenitor cells), and iPSCs (induced pluripotent stem cells), represent promising candidates for I/R injury repair. Despite animal models showing success, clinical replication remains challenging. To enhance the therapeutic efficacy, researchers utilize hypoxic conditioning, including pro-angiogenic activity and genetic modifications, chemical and physical surface modifications, and hydrogel encapsulation. Hypogenic preconditioning of MSCs and ADSCs significantly enhances VEGF levels, proliferation, and vessel density compared to normoxic MSCs [244,245]. Furthermore, stem cell-derived extracellular vesicles (EVs) can deliver microRNAs, such as miRNAs miR221-3p, ischemic tissues to promote angiogenesis and reduce apoptosis in a mouse model of MIRI [246]. The integration of RGD hydrogels with EVs enhances the retention and stability of HIF-1α engineered MSCs, significantly improving outcomes after acute MI [247]. These therapies modulate crucial survival pathways, including the suppression of autophagy via the p27 b/mTOR pathway [248] and PTEN/Akt axes [249]. These findings indicate that both gene and cell-based therapies hold potential strategies in complex surgical conditions; further efforts should also focus on developing dual HIF-1/2 inhibitors to improve therapeutic precision [250].

### 5.3. Preconditioning and Postconditioning Effects in I/R

Intermittent hypoxic preconditioning (IHP) has been shown to protect against doxorubicin-induced cardiomyopathy, aligning with its benefits in traditional I/R models. Although the mechanisms are not fully understood, IHP appears to reduce intracellular Ca^2+^ overload [251,252] and enhance the Ca^2+^ uptake by the sarcoplasmic reticulum, which improves heart contractility and limits myocardial injury. These protective effects may involve HIF-1α-dependent pathways, such as angiogenesis [253] and regulation of SERCA2a [254], both of which can be impaired by doxorubicin-induced suppression of HIF-1α [255]. Additionally, some protection may result from the activation of transient receptor potential vanilloid (TRPV) channels, which help restore NO-mediated signaling after IHP [256]. Overall, these findings highlight IHP as a promising non-pharmacologic strategy to counteract doxorubicin-induced cardiotoxicity through a coordinated mechanism involving HIF/TRPV–SERCA2a, warranting further translational research [257].

Ischemic preconditioning (IPC) has long been recognized for its ability to activate HIF-1α, leading to a reduction in the production of proinflammatory cytokines and a decrease in apoptosis within target tissues [258]. IPC also protects the myocardium by increasing iNOS expression in a HIF-1α-dependent manner [259]. Both remote IPC and late IPC enhance IL-10 levels, and these protective effects require HIF-1α expression, ultimately reducing the severity of MIRI [260]. For instance, in vivo studies have shown that remote ischemic preconditioning, induced by brief episodes of limb ischemia, provides protection from cardiac injury [261]. This protection is potentially mediated by HIF-dependent upregulation of serum interleukin-10 (IL-10), which results in a reduction in infarct size following myocardial infarction [260]. However, some studies report contradictory or minimal effects, indicating that the role of HIF-1α in remote ischemic preconditioning may vary depending on context and be influenced by factors such as the duration of ischemia, tissue type, and comorbid conditions [262]. These findings highlight the crucial role of HIF-1α in IPC and its significance as a key modulator of protection against myocardial I/R injury [263].

Ischemic postconditioning (IPostC) attenuates I/R injury by limiting ROS production, calcium overload, inflammatory response, and enhancing endothelial cell function [264]. Mechanistically, IPostC activates various pro-survival kinases, including PI3K [265], eNOS [266], ERK1/2 [267], GSK-3β [268], β-catenin, and reperfusion injury salvage kinase (RISK) pathway, like Akt/protein kinase B [269,270]. It occurs in two distinct phases: an immediate passive phase following the ischemic event and an active phase starting 24 h later. Although both the pre- and post-conditioning provide cardioprotective effects following I/R injury, the clinical applicability of preconditioning is often limited due to the unpredictable nature of ischemic events. In contrast, postconditioning is more feasible in a clinical setting, as it can be initiated at the onset of reperfusion after ischemia. Consequently, current research is focused on elucidating the molecular mechanisms underlying postconditioning-induced cardioprotective events, to translate these findings into effective and practical therapeutic strategies for alleviating I/R injury [271].

**Table 1:** Pharmacological modulators of HIFs’ functions in cardiomyopathy and I/R injury.

S. No.	Drug/Compounds	Molecular Mechanism	Clinical Applications	Reference
1.	Asiatic acid	Reduces ROS generation and improves mitochondrial function	Exhibits neuroprotective effects against ischemic injury and shows potential in attenuating neuronal damage	[239,272]
2.	Belzutifan (PT2977)	Selective inhibition of HIF-2α transcriptional activity by preventing HIF-2α/ HIF-1β dimerization	Approved after Phase III trials for advanced or metastatic clear cell renal cell carcinoma (ccRCC); anemia and hypoxia reported as adverse effects	[213,214,273]
3.	Dapagliflozin	SGLT2 inhibitor; suppresses HIF-1α/TGF-β–mediated cuproptosis	Improves glycemic control and blood pressure in T2DM; reduces risk of heart failure or CVD death in patients with mildly reduced or preserved ejection fraction	[209,210,274]
4.	Diltiazem (DIL)	Inhibits calcium overload by blocking L-type calcium channels	Reduces heart rate, blood pressure, and cardiac contractility; used in chronic angina, atrial fibrillation, and atrioventricular node control	[129]
5.	Daprodustat/Vadadustat/Molidustat/Enarodustat	PHD inhibition results in stabilization of HIF-1α	Enhances angiogenesis and erythropoiesis in PAD or chronic limb-threatening ischemia (CLTI)	[230,231,275]
6.	Dihydrotanshinone I (DT)	Inhibits mitochondrial Complex I and stabilizes HIF-1α	Enhances antioxidant defense, attenuates I/R injury through precondition, and protects against NLRP3 inflammasome-mediated inflammatory stress	[150,276,277]
7.	Dimethyloxalylglycine (DMOG)	Inhibits PHDs and FIH, stabilizes HIF-1α	Impairs excessive endothelial proliferation and mitigates ER stress in SCI	[224,278]
8.	Dexmedetomidine (DXE)	Highly selective α-2 receptor agonist; inhibits HIF-1α-induced BNIP3 expression and suppresses inflammation	Reduces myocardial infarction size during preconditioning; attenuates oxidative stress and inflammatory response	[218,219,279,280]
9.	Metformin	Activates AMPK signaling, inhibits NOX4 expression, reducing oxidative stress and apoptosis	Reduces infarction size in AMI in patient with T2DM; improves myocardial oxygen efficiency, glycemic control, and ejection fraction in heart failure	[217,281,282]
10.	Protocatechuic aldehyde (PCA)	Increases reduced GSH levels and enhances ROS scavenging	Alleviates endothelial dysfunction, prevents dyslipidemia, suppresses inflammatory markers, reduces plaque formation, and decreases infarct size during preconditioning	[150,282]
11.	Roxadustat (FG-4592)	PHD inhibitor, stabilizes HIF-α	Increases endogenous EPO levels, improves iron metabolism in CKD, and enhances renal and cardiac function	[221,283]
12.	Sevoflurane	Pretreatment upregulates VEGF expression	Reduces cardiomyocyte apoptosis, decreases ROS production, attenuates inflammation, and modulates pyroptosis	[220,284,285]
13.	Saponins	Induce mitophagy and inhibit NF-κB signaling	Ameliorate atherosclerosis, regulate lipid metabolism, exert anti-inflammatory effects, and prevent mitochondrial dysfunctions and fibrosis	[237,286,287,288]

## 6. Limitations of Pharmacological Modulation of HIFs in I/R

In response to hypoxia, the heart undergoes metabolic reprogramming, primarily regulated by HIFs, particularly through HIF-1α and HIF-β. In vivo models have shown that both the loss-of-function and gain-of-function mutations in the HIF-1α gene can lead to left ventricular pressure overload (LVPO) [289]; however, sex-specific differences in gene expression have been reported in LVPO [290]. Hypoxia has demonstrated cardioprotective effects in HF induced by LVPO, as observed in an HxTAC mouse model [291]. Under hypoxic conditions, HIFs trigger a series of cellular and functional adaptations in both myocardial and non-myocardial cells, significantly impacting metabolic signaling pathways and immune responses. Over the past three decades, numerous ischemic conditioning strategies and pharmacologic interventions have been explored to prevent I/R injury [292,293,294]. However, most monotherapy approaches aimed at treating myocardial injury have proven unsuccessful, as the factors involved in cardiac I/R injury are multifaceted and cannot be adequately addressed by a single treatment method [295].

The HIF stabilizer, DMOG, shows promise in treating acute renal failure and I/R injury in both in vivo and in vitro models without compromising endothelial cell number and function [296,297]. In spinal cord injury (SCI) models, DMOG promotes neuronal survival and axonal regeneration by reducing apoptosis and activating autophagy via the HIF-1α/BNIP3 signaling pathway [298]. These findings highlight DMOG’s multitargeted effects across different traumatic injuries. Similarly, the HIF-PHD inhibitor FG-2216 has demonstrated cardio-renal protection by improving nephropathy, cardiac function, and obesity in ZSF1 rats, a model used for kidney failure with metabolic syndrome [299]. Despite these beneficial effects, DMOG exhibits dose-dependent toxicity in PC12 cells (neuron-like cells), impairing mitochondrial activity and cell cycle progression [300]. In addition to these findings, a meta-analysis of chronic kidney disease patients found that HIF stabilization improves EPO synthesis, enhances iron mobilization, reduces hepcidin, and improves lipid profiles. This study showed no significant differences in CVD and mortality between the HIF stabilizer and the control group [301]. Thereby, these data indicate that these drugs’ effects on CVD remain limited and require further evaluation [302].

Further investigations into HIF-α signaling pathways and isoform-specific PHD inhibition may provide critical insights into the molecular mechanisms underlying I/R injury [303]. Specific mechanisms, including downstream targets and immune cell response, highlight promising targets following reperfusion. Most clinically available HIF-PH inhibitors affect all three isoforms of prolyl hydroxylase domain proteins (PHD1, PHD2, and PHD3), creating challenges in achieving specificity [303]. Notably, many studies have shown that isoform-specific activities, such as the inhibition of PHD2, offer cardioprotective benefits, while inhibiting PHD1 or PHD3 does not [304]. These findings emphasize the importance of selective targeting of PHD isoforms, particularly the inhibition of PHD2, which emerges as a more effective and promising strategy for enhancing protection from myocardial injury [305]. A limitation of pharmacological HIFs and HIF-PHD activators or inhibitors is their delayed onset of action. Therefore, direct activation or inhibition of HIF target genes may be more desirable, especially in acute MI conditions such as myocardial I/R injury. For instance, an immediate intervention is critical in a patient with acute MI. Combining HIF activation with an ADORA2B agonist may offer enhanced protection against myocardial I/R due to their rapid effects [306]. This suggests that ADORA2B agonists may promote more efficient recovery than HIF activators alone, as they exert faster cardioprotective effects [307,308]. Consequently, targeting the hypoxia–adenosine axis represents a promising strategy for preventing MIRI.

Over the past few decades, numerous small animal models have been developed, including experimental MI induced in rats and mice via left coronary artery ligation [309]. However, these MI/reperfusion models fail to capture the full clinical spectrum of patients, who often present with single or multiple risk factors [310]. Although studies have attempted to recapitulate the effects of metabolic syndrome, these models are unable to reflect complete human metabolic profiles [311]. Further, discrepancies in cardiac protein expression across the animal models limit their translational value [312]. For instance, mutations in key proteins such as cardiac phospholamban (PLN), δ-sarcoglycan (SGCD), RNA-binding protein 20 (RBP 20), and CRELD1 highlight the physiological gaps that restrict the clinical utility of these models [312].

## 7. Conclusions and Future Direction

HIFs are key regulators of oxygen homeostasis and play a significant role in various downstream pathways that impact the survival and function of cardiomyocytes [313]. Consequently, pharmacological inhibition of HIF-PH could offer therapeutic benefits for certain cardiovascular disorders, as well as other diseases such as chronic kidney disease, limb ischemia, inflammatory bowel disease, and cancer [314,315]. Specifically, ischemic preconditioning has been shown to help prepare heart muscles to better endure subsequent MIRI [306,316]. Both genetic and pharmacological studies indicate that HIF is involved in preconditioning and postconditioning in I/R. Notably, while HIF is implicated in several protective pathways, its activation may not be essential for all aspects of protection, as summarized in Table 2.

Additionally, regulation of downstream molecules, such as adenosine, can also confer benefits via purinergic signaling pathways [294,316]. Moreover, stimulation of the AKT/HIF-1α/VEGF pathways has demonstrated significant cardioprotective effects in both in vivo and in vitro models of I/R injury. These findings suggest that, in addition to HIF, targeting downstream factors may be beneficial for preconditioning and can enhance cardioprotective pathways. Additionally, remote preconditioning can protect both humoral and neural pathways, indicating that HIF also plays a role in this process [294,317]. Importantly, HIF-α signaling has dual and context-dependent effects, supporting both healthy cardiovascular homeostasis and the progression of cardiovascular diseases. Therefore, future research should aim to clarify these complexities, facilitating the safe and rational application of HIF-targeted therapies in clinical practice.

## Figures and Tables

**Table 2:** Summary of major pathways and human evidence supporting the roles of Hypoxia -ROS and HIF signaling in the pathogenesis of ischemic heart disease (IHD).

Pathways Linked with IHD	Key Characteristics	Human Evidence/Pathophysiological Roles	Major Signaling Pathways	References
**Hypoxia**	Insufficient oxygen supply to sustain normal cellular function, including metabolic reprogramming, cardiomyopathy, tumor progression, arrhythmogenesis, angiogenesis, and neurodegenerative disorders	Regulates HIF activity both in normoxic and hypoxic conditions; activates expression of genes including HO-1, EPO, GLUT1 and VEGF-A, and multiple glycolytic enzymes; impairs ion channel function and calcium overload, exacerbating cardiac injury; modulates inflammation and cell proliferation	PI3K/AKT, mTOR, NF-κB, ERK, Toll-like receptor (TLR), endoplasmic reticulum (ER) stress pathways	[24,67,68,70,89]
**Reactive Oxygen Species (ROS)**	Reduce oxygen conditions and activate mtROS production and Ca^2+^ overload, impairing ATP generation, fatty acid oxidation, and ketone metabolism, and inducing mPTP opening	Mitochondrial dysfunction promotes apoptosis and necrosis of cardiomyocytes during I/R, enhances Ca^2+^ release, induces HIF-1α/BNIP3 dependent mitophagy	PI3K/AKT, JAK2/STAT3, Nrf2, adrenergic and cholinergic signaling, canonical Gαs–cAMP–PKA pathway	[99,102,115,117,123,127,150]
**Hypoxia Inducible Factor (HIF)**	Identified by Semenza et al. in 1991 [41]; hypoxia stabilizes HIF expression. In contrast normoxia prevents its nuclear translocation via proteasomal degradation	During I/R injury HIF confers cardioprotection by activating purinergic signaling, enhancing angiogenesis, metabolic adaptation, and cell survival	iNOS signaling, Nrf2, NF-κB, AMPK pathways	[36,41,50,72,74,75,231]
**Therapeutic targets**	Strategies include HIF stabilization, PHD inhibition, antioxidant enhancement, reduction of mtROS levels, infarct size limitation, and apoptosis attenuation	Pharmacological and conditioning-based interventions improve cardiac outcomes: SGLT2 inhibitors, ischemic pre- and post-conditioning, IL-10 induction, mitochondrial autophagy activation, and ADORA2B agonists	ERK1/2, AKT, eNOS pathways	[51,197,198,209,218,231]

## Data Availability

No new data were created or analyzed in this study. Data sharing is not applicable to this article.

## Abbreviations

The following abbreviations are used in this manuscript:

4-HNE	4-hydroxy-2-nonenal
ACM	Arrhythmogenic cardiomyopathy
ARNT	Aryl hydrocarbon receptor nuclear translocator
βOHB	β-hydroxybutyrate
BBR	Berberine
CAD	Coronary artery disease
CTAD	C-terminal transactivation domain
CHD	Coronary heart disease
DCM	Dilated cardiomyopathy
DEX	Dexmedetomidine
DMOG	Dimethyloxalylglycine
DT	Dihydrotanshinone I
ER	Endoplasmic reticulum
EPO	Erythropoietin
ERK	Extracellular signal-regulated kinases
ETC	Electron transport chain
ETF	Electron transfer flavoprotein
NTPDase1/CD39	Ectonucleoside triphosphate diphosphohydrolase-1
FIH1	Factor-inhibiting HIF-1
GLUT1	Glucose transporter 1
H_2_O_2_	Hydrogen peroxide
HCM	Hypertrophic cardiomyopathy
HDAC	histone deacetylases
HF	Heart failure
HIFs	Hypoxia-inducible factors
HIF-PH	HIF-prolyl hydroxylase
HSP90	Heat shock protein 90
H/R	Hypoxia/reoxygenation
HO-1	Hemeoxygenase-1
IC	Ischemic cardiomyopathy
IHD	Ischemic heart disease
IHP	Intermittent hypoxic preconditioning
IPC	Ischemic preconditioning
IL-10	Interleukin-10
iNOS	Inducible nitric oxide synthase
I/R	Ischemia and reperfusion
IRI	Ischemic-reperfusion injury
IPAS	Inhibitory PAS protein
ISCU1/2	Iron-sulfur cluster enzyme ½
LV	Left ventricular
LVPO	Left ventricular pressure overload
MDA	Malondialdehyde
MI	Myocardial infarction
MIRI	Myocardial ischemia–reperfusion injury
mPTPs	Mitochondrial permeability transition pores
mTOR	Mammalian target of rapamycin
mtROS	Mitochondrial reactive oxygen species
NF-κB	nuclear factor-κB
NO	Nitric oxide
NOXs	NADPH oxidases
NOS	Nitric oxide synthases
O_2_^•−^	Superoxide
OXPHOS	Oxidative phosphorylation
PCA	Protocatechuic aldehyde
PHD	Prolyl hydroxylase domain
PKA	Protein kinase A
PI3K	Phosphoinositide 3-kinase
pVHL	Von Hippel–Lindau
ROS	Reactive oxygen species
RCM	Restrictive cardiomyopathy
SEV	Sevoflurane
SGLT2	Sodium–glucose cotransporter 2
SDH	Succinate dehydrogenase
SOD2	Superoxide dismutase
TRPV	Transient receptor potential vanilloid
T2DM	Type 2 diabetes mellitus
TCA	Tricarboxylic acid
TLR	Toll-like receptor
UPR	Unfolded protein response
VEGF-A	Vascular endothelial growth factor-A
WHO	World Health Organization
XO	Xanthine oxidase
